# Off-the-Shelf Allogeneic T Cell Therapies for Cancer: Opportunities and Challenges Using Naturally Occurring “Universal” Donor T Cells

**DOI:** 10.3389/fimmu.2020.583716

**Published:** 2020-11-11

**Authors:** Cynthia Perez, Isabelle Gruber, Caroline Arber

**Affiliations:** Department of Oncology UNIL CHUV, Ludwig Institute for Cancer Research Lausanne, Lausanne University Hospital and University of Lausanne, Lausanne, Switzerland

**Keywords:** allogeneic off-the-shelf T cells, virus-specific T cells, unconventional T cells, engineered, CD1, MR1, GVHD, rejection

## Abstract

Chimeric antigen receptor (CAR) engineered T cell therapies individually prepared for each patient with autologous T cells have recently changed clinical practice in the management of B cell malignancies. Even though CARs used to redirect polyclonal T cells to the tumor are not HLA restricted, CAR T cells are also characterized by their endogenous T cell receptor (TCR) repertoire. Tumor-antigen targeted TCR-based T cell therapies in clinical trials are thus far using “conventional” αβ-TCRs that recognize antigens presented as peptides in the context of the major histocompatibility complex. Thus, both CAR- and TCR-based adoptive T cell therapies (ACTs) are dictated by compatibility of the highly polymorphic HLA molecules between donors and recipients in order to avoid graft-versus-host disease and rejection. The development of third-party healthy donor derived well-characterized off-the-shelf cell therapy products that are readily available and broadly applicable is an intensive area of research. While genome engineering provides the tools to generate “universal” donor cells that can be redirected to cancers, we will focus our attention on third-party off-the-shelf strategies with T cells that are characterized by unique natural features and do not require genome editing for safe administration. Specifically, we will discuss the use of virus-specific T cells, lipid-restricted (CD1) T cells, MR1-restricted T cells, and γδ-TCR T cells. CD1- and MR1-restricted T cells are not HLA-restricted and have the potential to serve as a unique source of universal TCR sequences to be broadly applicable in TCR-based ACT as their targets are presented by the monomorphic CD1 or MR1 molecules on a wide variety of tumor types. For each cell type, we will summarize the stage of preclinical and clinical development and discuss opportunities and challenges to deliver off-the-shelf targeted cellular therapies against cancer.

## Introduction

Engineered T cell therapies using chimeric antigen receptors (CARs) against CD19^+^ B cell malignancies have been commercialized and have changed clinical practice. Current commercial products are manufactured in a highly personalized way for each individual patient with autologous peripheral blood αβ-TCR T cells (1, 2). Challenges with the use of autologous products include aspects related to previous chemotherapies or allogeneic hematopoietic cell transplantation (HCT) that can impact on the quantity and quality of the starting material, uncontrollable interpatient variability, and (too) long waiting times for the patients due to global manufacturing chains (3–5). Thus, the development of readily available off-the-shelf allogeneic immune effector cell (IEC) therapy products is an attractive alternative approach. Cell banks can be generated in advance; donors can be well-characterized according to the desired biological parameters of the final product. Major challenges to allogenic IEC therapies include the possibility of dual rejection: infused cells may produce graft-versus-host disease (GVHD), or the host immune system may reject the infused cells (4–6).

In this review, we will seek, evaluate and discuss challenges and opportunities for T cell-based IEC therapies, using naturally occurring “universal” donor T cells. These cells are either characterized by the recognition of well-defined HLA-restricted conventional αβ-TCR antigens, or are HLA-independent and recognize lipids, metabolites or phosphoantigens presented in the context of non-polymorphic receptors on target cells. By definition, these “universal” donor T cells do not produce GVHD, do not require genome editing for safe application as a therapeutic product, and have the capacity to potentially target a wide variety of cancers. We will focus our review on human preclinical and clinical developments including αβ-TCR T cells [virus specific T cells (VSTs), CD1-, and MR1-restricted T cells] as well as γδ-TCR T cells. Finally, we also discuss the potential use of universal TCRs that can be inserted as transgenes into IECs. Engineering of these “universal” donor T cells aims to combine and simultaneously exploit the endogenous natural properties of the cells with engineered properties that enhance the anti-tumor potential of the final product (e.g., recognition of tumor-derived lipids or metabolites by endogenous TCR and cell surface antigen by the introduced CAR).

## Naturally Occurring “Universal” Donor T Cells as Platforms for T Cell Engineering

### αβ-TCR T Cell Subsets

**Figure 1:**
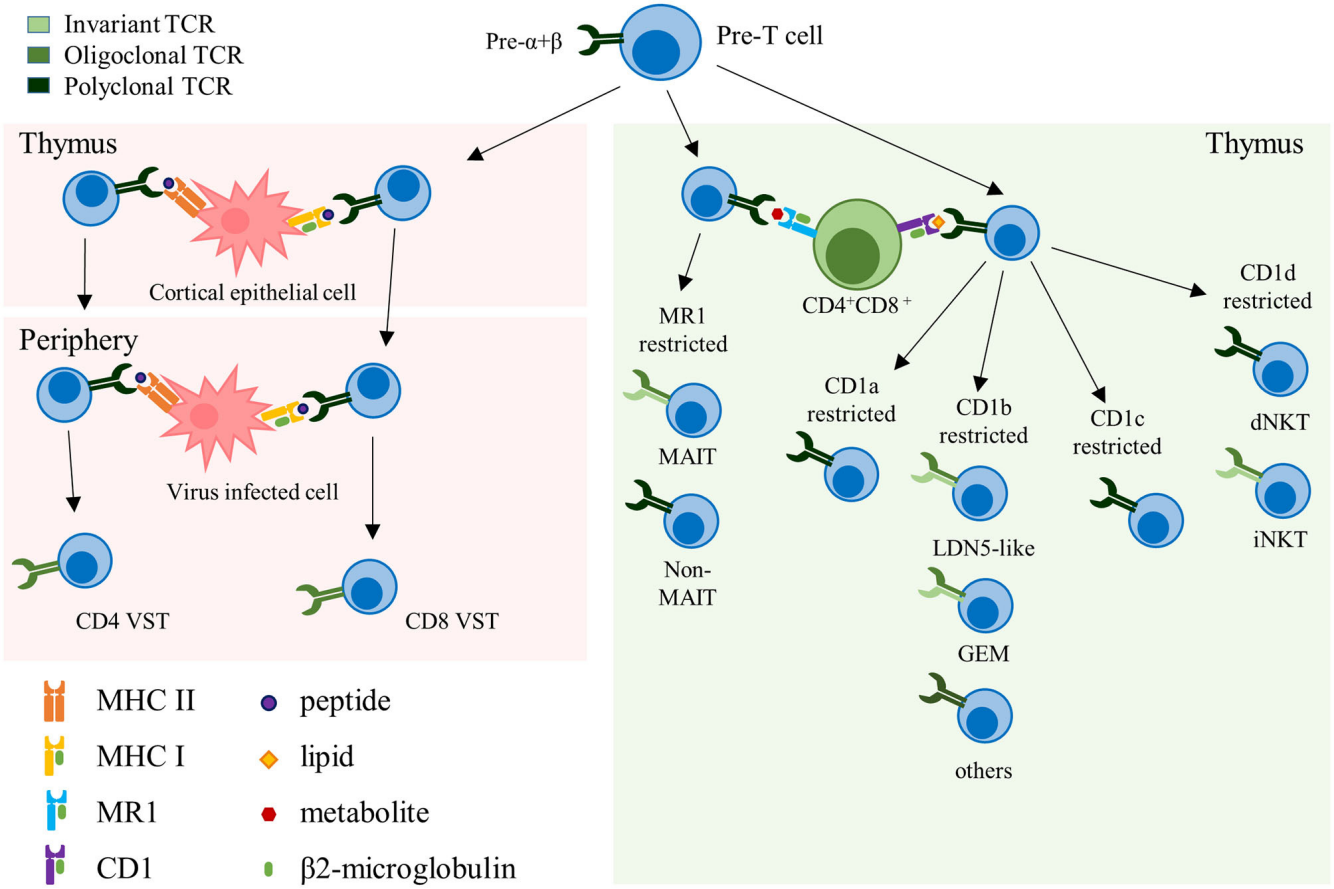
Representation of the different αβ-TCR subsets. Schematic representation summarizing the different αβ-TCR subsets, their development and restricting elements. The color grading of the TCR represents its clonality, from light to dark green (monoclonal to polyclonal, respectively). TCR, T cell receptor; VST, virus-specific T cell; MHC, major histocompatibility complex; MAIT, mucosal-associated invariant T cell; GEM, germline-encoded mycolyl lipid-reactive; dNKT, diverse natural killer T cell; iNKT, invariant natural killer T cell.

Conventional αβ-TCR T cells express HLA-restricted TCRs composed of an α- and a β-chain and recognize peptides presented by HLA molecules on the cell surface of target cells. Selected in the thymus, these T cells constitute the majority of the circulating T cells in the human body (7). Under physiologic conditions αβ-TCR T cells are polyclonal and express an extremely diverse TCR repertoire to cover a wide range of potential target antigens. This TCR diversity is reduced in memory T cell pools that form upon antigen specific expansion and clearance of a pathogen. VSTs directed against cytomegalovirus (CMV) for example are characterized by their oligoclonality, with a limited number of high avidity TCRs dominating the pool of memory VSTs that can re-expand upon repeated viral challenge (8–10). Unconventional αβ-TCR T cells are non-HLA-restricted and recognize non-peptide targets that are presented in the context of non-polymorphic molecules. In fact, several types of unconventional αβ-TCR T cells express semi-invariant TCRs, such as, for example, invariant natural killer T (iNKT) cells that recognize targets in the context of the monomorphic antigen-presenting molecule CD1, or mucosal-associated invariant T (MAIT) cells that recognize targets in the context of MR1 (Figure 1). Several of these αβ-TCR T cell subsets therefore harbor unique features that could potentially qualify them as universal donor cells for adoptive T cell therapy (ACT) (Table 1).

**Table 1:** Features of universal donor cells.

**Cell type**	**Size of the TCR repertoire**	**Polymorphism of restricting element**	TCR cross-pairing potential ^*^	**Difficulties of *ex vivo* expansion**	**Risk of off-tumor on target recognition**	**Reported or expected risk of GVHD**	**Universality score (lowest is best)**	**Intrinsic immune activity**	**References**
**αβ-TCR T cells**									
VST	++	+++	++	–	–	–	7	Anti-viral	(11, 12)
iNKT	+	+	+	–	+	–	4	Anti-tumoral, pro-inflammatory, protect from GVHD	(13–16)
dNKT	+++	+	+++	++	++	+	12	Immune suppressive	(7, 17, 18)
CD1a-restricted	+++	+	+++	+	++	+	11	Unknown	(19–21)
CD1b GEM	+	+	++	+	++	+	8	Unknown	(22, 23)
CD1b LDN5-like	+	+	++	+	++	+	8	Unknown	(22, 23)
CD1b-restricted	++	+	+++	+	++	+	10	Unknown	(19–21)
CD1c-restricted	+++	+	+++	+	++	+	11	Unknown	(19–21)
MAIT	+	+	+++	+++	++	+	11	Unknown	
**γδ-TCR T cells**									
Vγ9Vδ2	–	+	–	–	+	–	2	Anti-tumoral, pro-inflammatory, APC	(24–27)
Vδ1	+++	Unknown (non-HLA)	–	–	+	–	4	Anti-tumoral, pro-inflammatory	(28)

*Table summarizing the different features restricting the use of a T cell subset as universal donor cells. The amplitude of each restricting feature is depicted as none/extremely low (–), low (+), intermediate (++) and high (+++). The addition of each (+) is reported in the “Universality score” column, which represents the universal potential of each T cell subset, with a lower score corresponding to higher universality*.

* *Upon introduction of a transgenic TCR*.

*TCR, T cell receptor; VST, virus-specific T cell; MAIT, mucosal-associated invariant T cell; GEM, germline-encoded mycolyl lipid-reactive; dNKT, diverse natural killer T cells; iNKT, invariant natural killer T cells; HLA, human leukocyte antigen; GVHD, graft-versus-host disease; APC, antigen-presenting cell*.

#### Virus-Specific T Cells (VSTs)

##### Adoptive transfer of VSTs to prevent or treat infections and/or EBV-associated post-transplant lymphoproliferation

Viral reactivations and infections after allogeneic HCT remain a major cause of morbidity and mortality (29). Current anti-viral drugs are associated with dose-limiting end organ toxicities or lack of efficacy due to primary or secondary resistance, and only virus-specific immune reconstitution can resolve the issue of recurrent infections. Thus, in patients that do not concomitantly present with GVHD, adoptive transfer of VSTs is a safe and efficient therapy to accelerate immune reconstitution. The various approaches of VST manufacturing for adoptive transfer as well as clinical trial results have recently been reviewed (30–32). Allogeneic HCT donor derived VSTs have shown significant clinical activity against Epstein-Barr Virus (EBV), CMV, adenovirus, BK virus, and Human Herpes Virus 6. Importantly, impressive anti-viral responses have been reported across studies, and significant alloreactivity or GVHD has only been described in a very limited number of patients. HCT donor derived Epstein-Barr Virus specific T cells (EBVSTs) are also active against post-transplant EBV^+^ lymphoproliferative disorders in 65–85% of treated patients (11, 33, 34), and their long-term persistence was demonstrated in gene marking studies (11).

With the goal to facilitate rapid access to VSTs including for patients with seronegative donors, allogeneic third-party VST banks have been developed by several groups and institutions (12, 30, 35–37) and have entered commercialization. Safety and anti-viral activity of adoptively transferred allogeneic third-party donor VSTs are excellent, and the overall response rate (ORR) when treating viral infections can reach up to 92% (36, 38). When targeting EBV^+^ lymphoproliferation, ORRs were 68% in HCT and 54% in solid organ transplant (SOT) patients (12). Because persistence is a key parameter for tumor targeted T cell therapies but has not been summarized in the recent review articles on VSTs, we selected clinical trials with information on persistence on the infused third-party VSTs and summarized the results in Table 2. Available information is quite scarce. Each group used different tracking methods, no gene-marking was available to easily detect the *in vivo* fate of the infused cells, and long-term follow-up is mostly lacking. Available data suggests that third-party VSTs do not engraft and persist as well as HLA-matched HCT donor-derived VSTs. The variability between patients and trials was broad. Gallot et al. for example were not able to detect significant levels of cells derived from the infused VST lines (41). But others found a correlation between detection of anti-viral activity (12, 38, 39, 42) or VST line derived TCR sequences (37, 40) and viral clearance. The limited persistence is most likely due to rejection of the infused third party VSTs by the host immune system. Thus, in order to overcome this problem, novel types of chimeric alloimmune defense receptors (ADRs) have been developed. One strategy consists of a chimeric receptor using the extracellular part of human β2-microglobulin and signaling through CD3ζ and was shown to protect VSTs from alloreactive T cells *in vitro*, but this approach cannot protect from NK cell mediated rejection (43). More recently, a receptor recognizing 4-1BB (CD137)—temporarily upregulated on both activated T and NK cells—and signaling through CD3ζ was developed. 4-1BBζ ADR-engineered T cells were protected from T and NK cell mediated rejection *in vitro* and *in vivo* in a mouse xenograft model, and CARs retained their antitumor function when co-expressed with the 4-1BBζ ADR (44). Thus, ADRs have the potential to further enhance the persistence, efficacy, universality and safety of third-party engineered VSTs, and can be co-engineered with CARs. In the post-transplant setting, endogenous immune reconstitution also plays an important role in the establishment of long-term viral control.

**Table 2:** *In vivo* persistence of third-party off-the-shelf VSTs.

**Targeted virus/es**	**Treatment indication**	***N* patients/treatment**	**Persistence evaluated in *N***	**Technique of detection**	**Result/persistence**	**References**
EBV	EBV^+^ lymphoma	33 SOT	5	TCR spectratyping	Up to 7 days post-infusion Trace of infusion product detected in 3/5 patients analyzed	(37)
EBV	EBV^+^ lymphoma	2, HCT (cord blood)	2	CTLp by LD	No durable engraftment, but transient CTLp increase 7–10 days after infusion	(39)
CMV, AdV, EBV	Infection and EBV^+^ lymphoma	50 HCT, 9 with EBV^+^ lymphoma	6 (4 responders, 2 non-responders)	TCR Vβ CDR3 sequencing	Clones derived from the VST line detectable in 4 responders up to 12 weeks	(40)
EBV	EBV^+^ lymphoma	6 HCT 3 SOT 2 non-transplant	8	STR on PBMCs	Signal barely detectable in 3/8 patients, up to day 10	(41)
EBV, AdV, CMV, BKV, HHV6	Infection and EBV^+^ lymphoma	38 HCT, 1 with EBV^+^ lymphoma	16 responders	IFN-γ ELISPOT with informative epitopes (VST line, patient or shared origin)	11/16 (69%) persistence up to 12 weeks, HLA match at 2 or more alleles Confirmed by STR in 1 case	(38)
CMV	Infection	10 HCT	8	IFN-γ ELISPOT with informative epitopes	5/8 activity of infused VST line, 8/8 activity against shared epitopes between line and patient	(42)
EBV	EBV^+^ lymphoma	33 HCT 13 SOT	3 HCT 3 SOT	STR on *ex vivo* EBV restimulated T cells	HCT: 1: CR, 100% VST line derived (day 10) 1: SD, no VST line derived cells detected 1: CR, 100% VST line derived cells day 32 SOT: 1: no response, no persistence 1: durable PR, long-term persistence of VST line derived cells (24 months) 1: durable PR, no VST line derived cells but host reconstitution	(12)

*EBV, Epstein-Barr Virus; CMV, Cytomegalovirus; AdV, Adenovirus; BKV, BK Virus; HHV6, Human Herpes Virus 6; SOT, Solid organ transplant; TCR, T cell receptor; HCT, hematopoietic cell transplant; CTLp, cytotoxic T lymphocyte precursors; LD, limiting dilution; STR, short tandem repeat; PBMCs, peripheral blood mononuclear cells; IFN, Interferon; ELISPOT, enzyme-linked immunospot; CR, complete response; PR, partial response; SD, stable disease*.

We still need to learn more about the contributions of infused cells and endogenously reconstituted anti-viral immunity upon third-party VST adoptive transfer when targeting viral infections or EBV-associated malignancies. Systematic assessment of VST persistence with standardized methods across clinical trials would facilitate this understanding.

##### Engineered VSTs in clinical trials

VSTs have been clinically validated as a cellular therapy platform to genetically redirect antigen specificity against tumor-associated antigens (Table 3). With this approach, endogenous anti-viral TCR specificities can be exploited for the *in vivo* expansion and stimulation of the transgenic VSTs or targeting of viral-associated malignancies. Indeed, autologous CAR-engineered VSTs have been evaluated in clinical trials targeting GD2 for neuroblastoma (45, 46), and allogeneic HCT donor-derived CAR^+^ VSTs targeting CD19 in B-acute lymphoblastic leukemia (47–49). Reactivation of CMV in patients after allogeneic HCT and CD19 CAR^+^ CMVST infusion for example led to significant *in vivo* re-expansion of the infused cells and CD19 directed cytotoxic activity with elimination of B cells (49). To assess whether vaccination could be used to *in vivo* re-expand CAR^+^ VSTs, a clinical trial is underway to assess autologous GD2-CAR^+^ Varizella Zoster VST (VZVSTs) cell infusions in combination with vaccination (NCT01953900). Preclinical investigations had shown that anti-tumor function of GD2-CAR^+^ VZVSTs could be rescued *in vitro* upon stimulation with VZV peptide-pulsed dendritic cells (DC) (50). A clinical trial with gene-modified third-party partially HLA-matched healthy donor-derived banked CAR^+^ EBVSTs targeting CD30 in patients with EBV-associated CD30^+^ lymphomas is in preparation at Baylor College of Medicine (NCT04288726).

**Table 3:** Overview of clinical development status.

**Cell type**	**Frequency in circulating T cells**	**Isolation**	**GMP-compatible expansion**	**Genetic engineering**	Hypothetical number of cross-paired TCRs^*^	**Status of clinical translation**	**Reference**
**αβ-TCR T cells**							
VST	0.01–0.2%	Antigen specific expansion, IFNγ-capture, multimer selection	Yes	CAR and TCR	Low	In phase 1 and 2 clinical trials	(45–56)
iNKT	0.01–0.2%	α-GalCer-induced expansion	Yes	CAR and TCR	Low	In phase 1 clinical trials	(57–60)
dNKT	1%	Not done	No	Not done	High	Not in clinical trial	(61)
CD1a-restricted	0.1–10%	*In vitro* expansion with CD1-expressing cells	No	Not done	High	Not in clinical trial	(62)
CD1b GEM	<0.01%	Tetramer	No	Not done	Low	Not in clinical trial	(22, 23)
CD1b LDN5-like	<0.01%	Tetramer	No	Not done	Low	Not in clinical trial	(22, 23)
CD1b-restricted	0.1–10%	Single cell sorting for the generation of T cell clones	No	Not done	High	Not in clinical trial	(63)
CD1c-restricted	0.1–10%	Single cell sorting for the generation of T cell clones	No	Not done	High	Not in clinical trial	(63, 64)
MAIT	5–10%	Tetramer	No	Not done	Low	Not in clinical trial	(65–67)
**γδ-TCR T cells**							
Vγ9Vδ2	1–5%	Zoledronate-induced expansion	Yes	CAR and TCR	None	In phase 1 clinical trials	(68–70)
Vδ1	0.1–1%	Beads selection and cytokine expansion	Yes	CAR and TCR	None	Clinical trials expected soon	(71, 72)

* *Upon introduction of a transgenic TCR. TCR, T cell receptor; VST, virus-specific T cell; MAIT, mucosal-associated invariant T cells; GEM, germline-encoded mycolyl lipid-reactive; dNKT, diverse natural killer T cells; iNKT, invariant natural killer T cells; HLA, human leukocyte antigen; GVHD, graft-versus-host disease; APC, antigen-presenting cell; CAR, chimeric antigen receptor; GMP, Good Manufacturing Practice*.

Engineering VSTs with a tumor-targeted transgenic TCR has been more challenging than with CARs, as forced expression of a transgenic TCR leads to downregulation of the endogenous TCRs (51). Indeed, these findings were confirmed in TCR transgenic VSTs, where reduction of antiviral activity was reported in several preclinical studies (52–55) and in one clinical trial (56). Interestingly, one report showed that TCR^+^ VSTs can shift their antigenic predominance depending on the type of antigenic exposure given to the cells (viral or tumor antigen) (55). The oligoclonal features of VSTs minimize the risk of cross-pairing between transgenic and endogenous TCR chains, and thus the use of VSTs to express a transgenic TCR is thought to reduce the risk of both off-target toxicities and GVHD. In the only clinical trial reported to date, a Wilms tumor antigen-1 (WT-1) specific TCR was expressed in single epitope specific EBVSTs generated from the HLA-matched HCT donor. The clinical responses in high-risk AML patients who received allogeneic HCT followed by prophylactic WT-1 TCR^+^ EBVST infusions were impressive (56). Unfortunately, no viral reactivation occurred in the cohort of 12 treated patients, so the question whether the level of anti-viral specificity is sufficient to mediate *in vivo* re-expansion of TCR^+^ VSTs upon viral reactivation and to protect against viral disease remains elusive. TCR transgenic third-party VSTs have not yet been evaluated clinically.

##### VSTs as platform for engineered ACT: how universal can they be?

Third-party banked VSTs have been established as a safe and efficient ACT to treat infections and EBV^+^ lymphoproliferation after allogeneic HCT or SOT. The oligoclonal nature of VSTs limits their capacity to induce GVHD in this patient population. Despite the polymorphic nature of HLA, VST banks can be built with a limited number of well-chosen and characterized donors to cover a highly diverse patient population (42). Furthermore, VSTs can efficiently be redirected to tumors with both CARs and TCRs for clinical use. Drawbacks of third-party VSTs are that (i) their use has so far been limited to HCT and SOT patients, (ii) the *in vivo* re-stimulation through the endogenous TCR depends on unpredictable endogenous viral reactivations or scheduled vaccinations, and (iii) their long-term persistence has not yet been conclusively assessed. The clinical development of engineering strategies such as incorporation of ADRs may overcome these limitations in the future and make VSTs safer and more universal.

#### CD1-Restricted T Cells

##### Background on CD1 molecules

The monomorphic CD1 family is constituted of five members, four extracellular (CD1a-CD1d) and one intracellular molecule (CD1e). CD1a, CD1b, and CD1c belong to the group 1 CD1, while group 2 is solely constituted of CD1d (7, 19). Circulating within the different secretory and endosomal compartments of the cells, CD1 molecules present a large array of lipid antigens to T cells (7, 73–75). So far, only a limited number of CD1-restricted antigens are known, consisting of lipids shared by multiple microorganisms and of self-lipids that accumulate during cellular stress, for example in cancer (19, 20, 76). CD1d expression is constitutive and present on all antigen-presenting cells (APCs), while the expression of CD1a/b/c is inducible and limited to a subset of APCs. For example, B cells express CD1c, Langerhans cells express CD1a and CD1e, while myeloid cells can express all five CD1 molecules (19, 20).

##### CD1d-Restricted T cells: their implication in tumor immunity

CD1d-restricted T cells, also called NKT cells, are selected in the thymus after recognition of CD1d molecules expressed by CD4^+^CD8^+^ double positive thymocytes (13, 19, 77). CD1d-restricted T cells are classified into two distinct groups based on their ability to recognize α-galactosylceramide (α-GalCer), a glycosphingolipid originally derived from marine sponge. Type I NKT, or iNKT, express a semi-invariant TCR consisting of an invariant TCRα chain (TRAV10-TRAJ18 in human) paired with a limited number of TCRβ chains and recognize α-GalCer. Type II NKT, or dNKT, express a more diverse polyclonal TCR repertoire, and are unresponsive to α-GalCer [(7); Figure 1].

α-GalCer is produced by the gut flora and many mammalian tissues and acts as a potent iNKT cell stimulator (14, 78–80). Dysregulated lipid production in tumors is also a source of antigenic lipids capable of stimulating iNKT cells [e.g., GD3 and GM3 in melanoma (81, 82), or α-fucosylceramides in colorectal and pancreatic adenocarcinomas (83)]. Upon TCR engagement, iNKTs rapidly secrete high levels of cytokines (e.g., IFNγ, TNFα, IL4, IL13, and IL17) and lytic granules (granzymes and perforin), and upregulate killing receptors such as Fas ligand and TRAIL (17, 84). Thus, iNKTs are rapidly cytotoxic and strongly modulate the tumor microenvironment by direct targeting of CD1d^+^ tumors, tumor-associated macrophages and myeloid-derived suppressor cells (15, 85). Modulation of the immune response occurs by transactivating NK cells, licensing DCs and activating γδ-TCR T cells (16, 19, 86). This crosstalk leads to a strong activation of the endogenous adaptive immune system (87). That iNKTs play an important role in anti-tumor immunity is inferred from the facts that low frequency of iNKT cells in patients with hematologic or head and neck cancers correlated with poor prognosis (88–90), while higher iNKT cell infiltration in colorectal cancer correlated with longer survival (91). After allogeneic HCT, higher doses of iNKT cells contained in the graft were associated with protection from acute GVHD (92), and early donor-derived iNKT cell reconstitution post-transplant correlated with reduced acute GVHD and lower non-relapse mortality while maintaining graft-versus-leukemia effects (93).

dNKTs are less well characterized and thought to have a more immune regulatory role (17, 18, 94). In multiple myeloma for example, dNKTs have been involved in suppression of anti-tumor immunity in an IL-13-dependent manner (94). Potential immunotherapeutic applications for dNKT cells have been reviewed elsewhere (61).

##### Ex vivo expanded iNKT cells in clinical trials

Due to their biology, iNKT cells are an attractive cell type to investigate for cancer immunotherapy (Table 3). However, establishing GMP compatible *ex vivo* expansion protocols for iNKT cells has been a hurdle to broader development. To date, results from two clinical trials assessing the safety of adoptively transferred *ex vivo* expanded autologous iNKT cells in cancer patients were reported (95, 96). In a lung cancer trial, autologous iNKT cells from 6 patients were expanded in the presence of α-GalCer and IL2, reaching 0.1–25% iNKT cells in the final products. Infusions were safe but no significant clinical responses were seen (96). In a melanoma trial, autologous iNKT cells were sorted from PBMCs and expanded *ex vivo* for 6–8 weeks with anti-CD3 and IL2. Purity post-expansion ranged from 13 to 87%. The nine treated patients had only minimal or no evidence of disease at time of infusion and were not lymphodepleted. A clear correlation between iNKT cell infusions, immune parameters and outcome could not be established (95).

Meanwhile, *ex vivo* expansion methods have been refined and now allow genetic engineering of iNKT cells (57, 58). Dual targeting by harnessing endogenous and engineered properties of iNKT cells produced very promising pre-clinical results in neuroblastoma with GD2-CAR iNKT cells also incorporating transgenic IL15 (57, 59) and in lymphoma with CD19-CAR iNKT cells expanded in media containing IL21 (58, 60). Both approaches have started phase I clinical evaluation at Baylor College of Medicine. Safety of autologous GD2-CAR.IL15 engineered iNKT cells is evaluated in patients with neuroblastoma (NCT03294954). Since iNKT cells are not alloreactive and clinical studies suggest that iNKTs can suppress GVHD (92, 93, 97), a clinical trial with third-party allogeneic off-the-shelf iNKT cells genetically engineered to express a CD19-CAR and IL15 is underway to assess safety, *in vivo* expansion and persistence, and responses in patients with B-cell malignancies (NCT03774654).

##### CD1d-Restricted T cells as platform for ACT

iNKT cells have the ability to kill CD1d^+^ tumor cells and immune suppressive cells in the tumor microenvironment through direct cytotoxicity, but also modulate the immune response of NK cells and DCs through cytokine secretion, producing enhanced anti-tumor responses of conventional endogenous T cells (16, 19, 86). In addition to its anti-tumor activity, iNKT cells can protect the patient from developing GVHD after allogeneic HCT, as better iNKT cell recovery correlated with a reduced risk of GVHD (92, 93, 97). With their lack of HLA-restriction, semi-invariant TCR and protective potential against GVHD, iNKT cells possess several unique features required for universal donor cells (Table 1). Their *in vivo* persistence will need to be analyzed. The field is currently moving toward evaluating the safe use of iNKT cells from allogeneic third-party universal donors in engineered ACT (NCT03774654).

##### CD1a/b/c-Restricted T cells and their implication in tumor immunity

The current knowledge on T cells restricted to group 1 CD1 is limited, and mostly results from studies performed *in vitro* on human T cell clones (62). Recognizing diverse microbial and self-lipid antigens, group 1 CD1-restricted T cells are relatively abundant among circulating lymphocytes in healthy individuals (63), and the majority has a polyclonal TCR repertoire (19–21). Two subsets of CD1b-restricted T cells, the germline-encoded, mycolyl lipid–reactive (GEM), and the LDN5-like T cells, which recognize glucose monomycolate, a lipid antigen derived from *Mycobacterium tuberculosis*, express an invariant TCR (TRAV1-TRAJ9, and TRAV17-TRBV4-1, respectively) (22, 23).

Group 1 CD1-restricted T cells are thought to participate in immune surveillance of hematologic malignancies. Analysis of a limited number of patient samples revealed positivity for CD1c in 51% and CD1b in 54% of AML patients (*n* = 33), 71% of B-ALL samples expressed CD1c (*n* = 7), and 75% of pediatric T-ALL samples expressed CD1a and CD1b (*n* = 8) (64). Methyl-lysophosphatidic acid (mLPA) is a self-lipid antigen presented in the context of CD1c on hematological malignancies (64). T cell clones recognizing mLPA in the context of CD1c produced higher levels of IFNγ when stimulated with malignant than with normal hematopoietic cells. Intracellular accumulation of mLPA in tumor cells is thought to increase CD1c-restricted presentation of mLPA on the cell surface compared to normal cells (64), and therefore leading to differential recognition of malignant cells with mLPA-restricted T cell clones.

##### Group 1 CD1-Restricted T cells as platform for ACT

Despite expression of CD1c on APC, tumor-reactive T cells differentially recognized CD1c-restricted mLPA presented by tumor cells, suggesting that CD1c-restricted lipid antigens specifically accumulate in malignant cells but not normal APCs (64). If CD1c-restricted T cells are made amenable to genetic engineering, they could be an interesting population to investigate for ACT. Similar prospects apply to CD1a- and CD1b-restricted T cells, as both CD1a and CD1b expression is restricted to APCs (19, 20). Due to their polyclonal TCR repertoire, CAR engineering could be more straightforward than TCR engineering due to potential cross-pairing of transgenic and endogenous TCR chains and higher risk to produce off-target toxicities.

CD1b-restricted invariant TCR T cells (GEM and LDN5-like T cells) theoretically are top candidates as universal donor cells for both CAR and TCR-based ACT (Table 1). However, their frequency is extremely low in *M. tuberculosis* positive patients [<0.01% of peripheral blood T cells (22)], and has not yet been described in healthy donors.

#### MR1-Restricted T Cells

##### General definition

The MR1 molecule is an evolutionary conserved, monomorphic protein (98, 99). Ubiquitously expressed, MR1 cell surface expression is however modulated by antigen abundance. Under physiological conditions, MR1 is almost undetectable at the cell surface. Bound antigen is needed for its trafficking to the cell surface, and cell surface expression is further enhanced by exogenous antigen loading (19, 98, 100). Known to be involved in the immunity against bacterial and yeast infections (101, 102), MR1 presents small metabolites derived from the metabolic pathways of vitamin B9 (folate) or B2 (riboflavin) (103). Only a few antigens have been identified so far, but the list is growing, and includes small cyclic molecules utilized as pharmacological agents, such as the aspirin analog 3-FSA (3-formylsalicylic acid) or the non-steroidal anti-inflammatory drug diclofenac, which suggests that MR1 may be involved in drug hypersensitivity (104, 105). Even if self-derived MR1-restricted antigens have not been identified yet, several studies suggest that such antigens exist (106). For several years, mucosal-associated invariant T cells (MAIT) were the only known T cells with MR1 restriction. Now there is growing evidence that MR1-restricted non-MAIT cells exist (107–109), but much more needs to be learned.

##### MR1-Restricted MAIT cells

MAIT cells develop upon interaction with CD4^+^CD8^+^ double positive cortical thymocytes, and continue to mature and expand after leaving the thymus [(105, 110, 111); Figure 1]. In humans, their numbers continuously increase during the first 25 years of life, and then slowly decrease with age (112, 113). Their expansion is thought to be dependent on stimulation with microbial antigens, as germ-free mice do not have any MAIT cells in the periphery, despite positive selection in the thymus (106).

Originally identified in the gut, MAIT cells are characterized by the expression of a semi-invariant TCR, constituted of an invariant TCR Vα chain (Vα7.2-Jα3.3) paired with a limited number of Vβ chains. While byproducts of the microbial riboflavin biosynthesis, such as 5-OP-RU (5-[2-oxopropylideneamino]-6-D-ribitylaminouracil), are known to strongly activate MAIT cells, the folate biosynthesis pathway generates molecules, such as 6-formyl-pterin, that exert inhibitory effects on MAIT cells (103, 114). Although MAIT cells constitute only 5% of the total T cell pool in humans, their frequency can greatly vary in different organs (105). MAIT cells are abundant in the liver, lung and gastro-intestinal tract, as well as in the blood. In the liver, for example, 45% of resident T cells are MAIT (115). In the periphery, MAIT cells constitute up to 10% of the circulating T cells (112). Similar to conventional T cells, MAIT activities can be modulated by the antigen recognized, the cytokines present in the microenvironment and the tissue to which they naturally home. In the colon, for example, MAIT cells preferentially display a Th1-type of cytokine secretion, and reside in the lamina propria and the intraepithelial compartment of the mucosa, while in lung and liver MAIT cells resemble tissue-resident memory T cells (116).

TCR engagement together with co-stimulation leads to rapid MAIT cell activation, in a memory-like manner (117, 118). Activated MAIT cells display both direct and indirect cytotoxic functions, through the secretion of granzyme B, perforin, and a large range of Th1 and Th17 type of cytokines (105). Together with their ability to home to infected sites, MAIT cells constitute an important player in anti-microbial defense.

##### MAIT cells in tumor immunity

MAIT cells are part of the tumor infiltrating lymphocyte population in colorectal cancer (116, 119, 120), hepatocellular carcinoma (121, 122), or kidney and brain tumors (123). In some tumors, an inverse correlation between circulating and tumor infiltrating MAIT cells was observed, but it is not clear yet if tumor-infiltrating MAIT cells are pro- or anti-tumorigenic. In brain and kidney tumors, for example, MAIT cell infiltration was associated with higher levels of pro-inflammatory cytokines (123). On the other hand, MAIT cell infiltration in colorectal cancer and hepatocellular carcinoma was associated with unfavorable clinical outcome. Failing to produce pro-inflammatory cytokines such as IFNγ upon *ex vivo* stimulation, these tumor-infiltrating MAIT cells were functionally impaired (119, 124). One study even showed by transcriptome sequencing analyses that MAIT cells infiltrating hepatocellular carcinoma acquired a pro-tumorigenic phenotype (122). Surprisingly, peripheral MAIT cells seem to be unaffected and retain their ability to respond to bacterial antigens and even to tumor cells (116, 120, 124).

In patients after allogeneic HCT, robust peripheral blood MAIT cell reconstitution has been associated with a lower risk for the development of subsequent severe acute GVHD (118, 125), and activated MAIT cells suppressed proliferation of CD4^+^ T cells *in vitro*. Correlations between gut microbiota composition, the related riboflavin pathway, and MAIT reconstitution exist (118, 126). Further investigations are necessary to decipher the precise role of MAIT cells in human GVHD and whether adoptively transferred *ex vivo* expanded MAIT cells could be immune suppressive.

##### MAIT cells as platform for ACT

MAIT cells possess unique features that would make them interesting candidates as universal donor cells for ACT (Table 1), and methods for their isolation and *ex vivo* expansion are being established (65–67). However, their precise role in anti-tumor immunity remains to be defined in more detail. Some of the burning questions include (i) if it is possible to reprogram them *in vitro* to express a stable Th1 profile, (ii) if adoptively transferred MAIT cells efficiently migrate to the tumor site and retain their anti-tumor function, and (iii) if they can be genetically engineered during *ex vivo* expansion. We are convinced that these and more questions will be answered soon, and MAIT cells will be investigated as universal donor cells.

### γδ-TCR T Cell Subsets

γδ-TCR T cells constitute 1–5% of total circulating T cells (14, 127). Selected in the thymus, the rearrangement process of γδ-TCR is highly complex and not fully understood yet. Constituted of a far smaller number of gene segments than αβ-TCRs, only 4–6 functional Vγ and 8 Vδ vs. 46 Vα and 48 Vβ chains, the potential of γδ-TCR diversity is however thought to surpass the diversity of αβ-TCRs (127, 128). Vδ4-Vδ7 gene segments rearrange with segments of the TCR α-chain, and have alternative TRAV names. Vδ1-Vδ2 preferentially rearrange with Dδ, Jδ, and Cδ to create TCR δ-chains, though few reports showed that Vδ1 and Vδ3 could also rearrange with segments of the TCRα locus, thus generating a δ/αβ-TCR T cell (129–132).

Vδ usage pre-determines γδ-TCR T cells function and localization: the majority of peripheral γδ-TCR T cells expresses Vδ2, while tissue-resident γδ-TCR T cells favor Vδ1 and Vδ3 (127). In humans, the majority of γδ-TCR T cells consists of Vδ1 and Vδ2 T cells (129). Their ligands are not well-characterized. The Vγ9Vδ2 T cell subset recognizes phosphorylated antigens, presented by the butyrophilin (BTN) molecules. Vδ1 γδ-TCR T cells can recognize antigens presented on CD1c, CD1d, and MR1 molecules. Both Vδ1 and Vδ2 γδ-TCR T cells are able to recognize stress-related molecules such as MIC A/B either via their TCR or via NK receptors such as NKG2D (129, 133, 134).

#### BTN-Restricted Vγ9Vδ2 T Cells

##### General background

The Vγ9Vδ2 T cell subset is relatively abundant in circulating lymphocytes, and represents 1–5% of all T cells in healthy individuals, and 50–95% of γδ-TCR T cells (127, 135, 136). This T cell subset expresses an invariant TCR that recognizes phosphorylated isoprenoid metabolites, also called phosphoantigens, derived from the mevalonate pathway. These metabolites, such as the isopentenyl pyrophosphate (IPP), can accumulate in transformed and infected cells because of their dysregulated metabolism (127, 135, 137). IPP accumulates when the activity of the IPP-metabolizing enzyme farnesyl-diphosphate-synthase (FPPS) is blocked (135). The use of aminobiphosphonates, such as zoledronate, inhibits FFPS, which leads to an increase in intracellular level of IPP and the activation of Vγ9Vδ2 T cells (135, 138–140).

IPPs are presented to T cells by butyrophilin molecules. Belonging to the immunoglobulin superfamily, these glycoproteins are divided into three subfamilies (BTN1, BTN2, BTN3) (141). Only BTN3A (CD277) presents phosphoantigens to Vγ9Vδ2 T cells. Constituted of three isoforms, BTN3A molecules are expressed by the majority of human immune cells, including γδ-TCR T cells (142). Phosphoantigens bind the intracellular domain B30.2 of BTN3A, which induces conformational changes to the receptor and increases binding force of Vγ9Vδ2 TCR to BTN3A (129, 143, 144). Therefore, Vγ9Vδ2 T cells are able to recognize altered metabolites present in infected or cancer cells.

##### Vγ9Vδ2 T cells in tumor immunity

Once activated, Vγ9Vδ2 T cells acquire similar effector functions as conventional αβ-TCR T cells: they directly kill tumor cells upon engagement of death receptors (e.g., FAS, TRAIL, NKG2D) or by secreting granzymes and perforins (24). In addition, Vγ9Vδ2 T cells produce various pro-inflammatory cytokines such as TNFα or IFNγ, and can modulate the immune response. For example, Vδ9Vδ2 T cells can license and accelerate DC maturation (145, 146) and provide help to B cells (147).

Human γδ-TCR T cells can be expanded *in vitro* to clinically relevant numbers, are able to migrate to and kill tumors, and are amenable to genetic engineering (68–70, 148, 149). Both HLA class I- and class II-restricted αβ-TCRs have been successfully introduced into Vγ9Vδ2 T cells and recognized the cognate peptide when co-transduced with CD4 or CD8 co-receptors (69, 70). TCR mispairing between α-/β-chains and γ-/δ-chains cannot occur. Thus, γδ-TCR T cells are optimal recipients for transgenic αβ-TCRs. αβ-TCR-transgenic γδ-TCR T cells express both αβ- and γδ-TCRs and mediate tumor cytotoxicity through both TCRs (69). Vγ9Vδ2 T cells were also engineered to transiently express a TCR or a CAR and exerted both endogenous and engineered properties (68).

One unique feature of Vγ9Vδ2 T cells is their capacity to differentiate into professional APCs upon IPP stimulation. Vγ9Vδ2 T cells can phagocytose cells and crosspresent antigens, leading to the proliferation of both CD4^+^ and CD8^+^ T cells (25, 26). This interesting feature is maintained in genetically engineered Vγ9Vδ2 T cells. For example, GD2-CAR-transduced Vγ9Vδ2 T cells killed GD2^+^ neuroblastoma, while retaining their ability to endocytose long peptides derived from the melanoma antigen MART-1 and inducing the proliferation of autologous T cells transduced with a MART-1-specific TCR (27).

#### Non-Vδ2 γδ-TCR T Cells

##### General background

Non-Vδ2 γδ-TCR T cells consist of Vδ1 and Vδ3 γδ-TCR T cells, and are mostly tissue-resident T cells present in barrier epithelium, though some of these cells are also circulating in blood (127). Between these two subsets, Vδ1 are the most abundant. From the original diverse repertoire present in neonates, only few Vδ1 γδ-TCR T cell clones will expand and ultimately dominate the adult Vδ1 repertoire (150). Even though the antigens recognized by Vδ1 γδ-TCR T cells are mostly unknown, they were shown to recognize ligands presented by CD1a, CD1c, CD1d, and MR1 molecules as well as various stress-induced ligands (133, 151, 152).

##### CD1-Restricted Vδ1 γδ-TCR T cells

CD1 molecules were among the first ligands identified for γδ-TCR T cells (153), but only few CD1-restricted lipids recognized by Vδ1 γδ-TCR T cells have been identified so far. Exogenous antigens comprise pollen-derived phospholipids and bacterial lipids, while known self-lipids consist of glycolipid sulfatides, which are present in tissues where Vδ1 γδ-TCR T cells reside (154–157). γδ-TCR T cells have been reported to be involved in tissue repair and homeostasis (158, 159), and predominate among γδ-TCR T cells that infiltrate various tumors (see below). However, the presence of CD1-resticted γδ-TCR T cells in tumors, and their involvement in tumor immunity, have not been investigated yet.

Crystal structure of CD1d-Vδ1 binding reveals a distinct TCR recognition: CD1d recognition by Vδ1 γδ-TCR T cells is solely mediated by the germline-encoded CDR1 loop, independently of the bound antigen. The antigen is in contact with the CDR3 region, which determines the antigen specificity (156, 160). Similarly, CD1c recognition was also shown to be dictated solely by Vδ1. Bound antigens modulate TCR recognition: some self-lipids were shown to permit TCR binding, while other self-lipids blocked it (154).

##### MR1-Restricted Vδ1 γδ-TCR T cells

γδ-TCR T cells recognizing antigens presented by MR1 have only been recently identified (152). This novel γδ-TCR T cell subset is rare with a frequency between <0.001 and 0.1% of total CD3^+^ T cells, or <0.1–5% of γδ-TCR T cells in blood of healthy donors, but has also been found in TILs of a Merkel cell carcinoma patient. MR1-restricted γδ-TCR T cells preferentially expressed Vδ1 (72% of the 76 TCRs analyzed). Structural studies revealed that TCR recognition occurred by binding to the MR1 α3 domain situated underneath the antigen-binding site and independently of the bound antigen (152) suggesting inherent autoreactivity of these cells.

##### Vδ1 γδ-TCR T cells in tumor immunity

Consistent with their preferential localization in epithelial tissues, Vδ1 γδ-TCR T cells are the predominant γδ-TCR T cell population in the majority of solid tumors (28, 161–163). Vδ1 γδ-TCR T cell infiltration correlated with favorable prognosis in several cancer types, such as triple negative breast cancer (TNBC) or CLL (162, 164). Upon *in vitro* expansion, Vδ1 γδ-TCR T cells isolated from PBMCs of cancer patients displayed strong IFNγ secretion and cytotoxic responses against several autologous tumors including melanoma (165), TNBC (162), colon cancer (166), AML (71), CLL (164, 167), diffuse large B cell lymphoma (168), and multiple myeloma (169). *In vitro* expanded Vδ1 γδ-TCR T cells were able to kill autologous CLL *in vitro* and spare healthy B cells isolated from the same patient, thus showing their ability to distinguish transformed cells from healthy cells (164). Moreover, these Vδ1 γδ-TCR T cells restrained tumor growth and prolonged the survival of immunodeficient mice engrafted with either human colon cancer or AML (71, 166). In other tumors, such as breast cancer (161, 170), colorectal cancer (163), melanoma (171), or squamous cell carcinoma (172), Vδ1 γδ-TCR T cell infiltration was associated with poor prognosis, as these cells displayed an immunosuppressive phenotype promoting tumor growth (170, 171). Vδ1 γδ-TCR T cells were shown to differentiate into Th17-like T cells, producing elevated level of IL-17, and other immunosuppressive factors, such as IL-10, IL-18, and adenosine (161, 170). Cancer cells were directly responsible for the skewing of Vδ1 γδ-TCR T cells toward Th17 regulatory profile. Breast cancer, for example, secretes exosomes containing the IncRNA SNHG16, a long non-coding RNA inducing the expression of SMAD5, and therefore TGF-β1, in Vδ1 γδ-TCR T cells (161). In colorectal cancer patients, cancer stem cells directly promoted IL-17 production by Vδ1 γδ-TCR T cells by secreting immunomodulatory molecules. By multiplex analyses on 50 different cytokines, the authors identified IL-18 and VEGF as the two most promising candidates responsible for the skewing of Vδ1 γδ-TCR T cells toward IL-17-producing immunosuppressive cells (163).

So far, no clinical trials have investigated the safety and efficacy of Vδ1 γδ-TCR T cells as anti-tumor therapy (Table 3). However, several groups established GMP-compatible protocols to expand and genetically engineer Vδ1 γδ-TCR T cells *in vitro* with the goal to translate this approach to the clinic (27, 28, 71, 72). Vδ1 γδ-TCR T cells can be expanded from healthy donor or patient PBMCs using a cocktail of different cytokines and anti-CD3 antibody stimulation. Over 3 weeks of culture, cells expanded more than 3-log fold and differentiated into cytotoxic Th1-like T cells, capable of controlling tumor growth in xenograft mouse models (28, 71). Moreover, Vδ1 γδ-TCR T cells expanded from PBMCs could be transduced with an anti-GD2 CAR and killed GD2-positive neuroblastoma cells lines that were not naturally recognized by Vδ1 γδ-TCR T cells (27).

#### γδ-TCR T Cells in Clinical Trials

Published and ongoing clinical trials investigating the safety and antitumor function of γδ-TCR T cells have recently been reviewed (173). Overall, the results of published trials outside the setting of allogeneic HCT have been disappointing, demonstrating safety but no efficacy. A major limitation lies in the lack of understanding of γδ-TCR diversity and their potential target antigens (Table 3). Some of the current and future clinical trials are trying to address these issues. We will focus our discussion on efforts in developing allogeneic γδ-TCR T cell therapies, with the overall goal to move their application to third-party banked off-the-shelf therapies. We identified five registered clinical trials, but more are expected to emerge. Three trials explore the safety of allogeneic *ex vivo* expanded adoptively transferred γδ-TCR T cells in hematologic malignancies. In one trial, γδ-TCR T cells are expanded from the haploidentical stem cell donor and infused to the patient post-transplant in combination with post-transplant cyclophosphamide treatment (NCT03533816, Incysus Therapeutics). Safety and effects on post-transplant GVHD are investigated. Another trial assesses the adoptive transfer of *ex vivo* expanded γδ-TCR T cells derived from related haploidentical or HLA-matched donors in patients with relapsed/refractory AML after lymphodepleting chemotherapy (NCT03790072, TC Biopharm). Safety and efficacy are analyzed. Future prospects are to move to allogeneic third-party banked γδ-TCR T cell products, and to include genetic engineering with CARs. A third active trial is also investigating *ex vivo* expanded γδ-TCR T cells from allogeneic related donors in patients with relapsed/refractory AML (NCT04008381, Wuhan Hospital). For solid tumors, a randomized clinical trial investigates safety and efficacy of tumor reducing surgery alone or in combination with adoptive transfer of *ex vivo* expanded γδ-TCR T cells in patients with locally advanced pancreatic cancer. The source of the γδ-TCR T cells is not entirely clear (NCT03180437, Fuda Cancer Hospital). Finally, haploidentical NKG2DL-CAR engineered γδ-TCR T cells for a variety of relapsed or refractory solid tumors will be investigated in a phase I dose escalation trial (NCT04107142, Cytomed Therapeutics).

#### γδ-TCR T Cells as Platform for ACT

γδ-TCR T cell expansion protocols allow the incorporation of genetic engineering to redirect γδ-TCR T cells to tumor-associated antigens recognized by CARs or αβ-TCRs (28, 68, 71, 72). Preclinical data suggest that γδ-TCR T cells are particularly suitable for αβ-TCR-based engineering, as the risk of TCR mispairing is inexistent. Both TCRs remain well-expressed, and the redirected T cells can exert anti-tumor functions through both TCRs. As γδ-TCR T cells are not HLA restricted (137), there is theoretically no risk of causing GVHD in the recipients, but formal demonstration in a clinical trial with third-party donor derived γδ-TCR T cells is currently lacking. γδ-TCR T cells meet many features required for universal donor T cell therapies (Table 1), and the interest in the field of exploiting this cell type is high.

## Universal TCR

TCRs derived from both αβ- and γδ-TCR repertoires that allow the targeting of a broad range of tumors in an HLA independent manner have been identified. These TCRs can be considered “universal,” as they redirect immune cells to broadly shared tumor-specific antigens. TCRs recognizing targets derived from altered cell metabolism are of particular interest, as these TCRs reliably distinguish between cancer and healthy cells. Three examples from the literature include Vγ9Vδ2 TCRs (174, 175), mLPA-specific CD1c-restricted αβ-TCRs (64), and an MR1-restricted αβ-TCR with unknown specificity (108). These TCRs have been successfully introduced into polyclonal αβ-TCR T cells and were able to redirect the engineered cells to a variety of cancers in an HLA-independent manner in preclinical studies. Autologous Vγ9Vδ2-TCR engineered αβ-TCR T cells are currently under phase I clinical evaluation in patients with hematologic malignancies (NTR6541, UMC Utrecht). More TCRs with similar features are likely to be identified in the future. TCRs targeting cancer-specific ligands in the context of non-polymorphic molecules are likely to become interesting candidates for engineered ACTs.

## Conclusions and Future Outlook

VSTs and unconventional T cells possess several features that would enable their universal use without the need of genome editing to avoid unacceptable alloreactivity. Among these different T cell subsets, γδ-TCR T cells, especially Vγ9Vδ2 T cells, and iNKT cells show the highest universal potential (Table 1). However, whether or not host-mediated rejection of the infused cells will affect their engraftment and long-term persistence remains to be addressed in the upcoming clinical trials. The only data available to date on persistence, safety, and efficacy come from third-party banked VSTs where, despite excellent clinical activity, persistence seems to be reduced compared to other trials that used HLA-matched products. Characterized by a memory-like status, VSTs and unconventional T cells respond rapidly to antigen exposure, leading to strong cytolytic activity, and cytokine production (19). Several of these T cell types have successfully been redirected to tumors by genetic engineering with a CAR or a TCR and mostly retain their intrinsic characteristics. For example, CAR^+^ VSTs maintain their anti-viral responses (48, 49), γδ-TCR T cells express both endogenous and introduced αβ-TCR (69), and CAR^+^ iNKT cells continue to respond to α-GalCer (57, 59, 176). The retention of these cell-specific features can also promote their *in vivo* re-expansion after ACT through re-exposure to their natural cognate antigen (49). Another advantage is the relatively restricted pattern of target antigens recognized by their endogenous TCRs. CD1-restricted and γδ-TCR T cells, for example, recognize ligands derived from altered tumor metabolism, and thus spare the corresponding healthy cells (36, 43). Despite still limited understanding of the biology of certain unconventional T cell subsets, the developing clinical translational pipelines outlined in this review demonstrate that the future potential for some of these experimental therapies as off-the-shelf products is high (Table 3). We expect that some of these cell types or universal receptors will become important players in the field cancer immunotherapy.

## Author Contributions

CP and CA: concept and writing of the manuscript. IG: preparation of the figure. All authors have read and agreed with the final version of the manuscript.

## Conflict of Interest

All authors declare that the research was conducted in the absence of any commercial or financial relationships that could be construed as a potential conflict of interest.
